# Dr. C. F. Taylor’s Spinal Apparatus

**Published:** 1866-02

**Authors:** F. O. Earle

**Affiliations:** 75 West 26th St.; New York


ARTICLE VI.

### DR. C. F. TAYLOR'S SPINAL APPARATUS.

To N. S. DAVIS, M.D., *Editor Chicago Med. Examiner.*

DEAR SIR:—In the paper on Orthopædic Surgery, by Dr. DAVID PRINCE, of Jacksonville, Ill., which appeared in the August number of your journal, the doctor, in alluding to the instrument used by Dr. CHARLES F. TAYLOR, of this city, for the treatment of Pott's Disease of the Spine, says:—

"The hinges in Dr. Taylor's apparatus must certainly be useless complications of machinery, for the very object of the apparatus is to obviate movements of the vertebræ upon each other, and not to make special provision for motion. The elasticity of the material affords all the motion which should be permitted."

Dr. PRINCE cannot have a right conception of the form and uses of the instrument, or he would never have made such a statement. He seems to think that the hinges allow of motion forward, in which case they would certainly be worse than useless bits of mechanism. If he will look on page 26 of "The Mechanical Treatment of Angular Curvature," he will learn that they are "stop hinges in front, but free to bend backwarks, and allow the most unrestrained use of the muscles of the back," but *only in one direction*, namely, *backwards*, "thus stimulating and encouraging muscular action, as the patient, when free to do so, involuntarily makes frequent efforts to gain momentary relief from the instrument, by attempting to straighten himself up. Indeed, the spinal muscles, by alternate action and rest, actually alternate with the instrument, in sustaining the weight of the body and overcoming the curvature."

"It has proved to be useful in causing the development of the spinal muscles, instead of binding them up and causing their atrophy, as results from the use of instruments which prevent muscular action."

But besides *preventing* motion forward, the hinges are of great advantage in another way. By referring to page 29 of Dr. Taylor's monograph before mentioned, we learn that:—"There are also little screws by which the hinges are forced open and the form of the instrument made straighter or more bent, as the case may be; thus increasing or diminishing the sustaining force at will."

Thus it will be seen that the hinges are used to *prevent* the motion which the "elasticity of the material" is sure to afford to the "vertebræ (*forward* by the weight of the body) *upon* each other," and that the only motion possible in Dr. Taylor's appliance (*backward*) is that which diminishes the pressure, by tending to bring the vertebræ *away* from each other.

During the past two years, I have enjoyed the opportunity of seeing quite a number of cases under treatment by Dr. Taylor's method, and have been struck by the simplicity and effectiveness of the instrument employed by him, and also by its easy adjustability and adaptation to the requirements of every case, and this knowledge of its superiority over every other that I have ever seen, has induced me to correct the erroneous views entertained by Dr. PRINCE regarding this very useful surgical appliance. Very respectfully,

F. O. EARLE, M.D.,

*New York, Dec. 28th,* 1865. 75 *West* 26*th St.*

